# Accelerated Discovery of Earth‐Abundant Oxygen Evolution Catalysts Through Surface‐Informed Active Learning

**DOI:** 10.1002/advs.78281

**Published:** 2026-10-22

**Authors:** Hayun Jeon, Minhee Park, Won Bo Lee, Seyoung Park, YongJoo Kim, Sungwook Mhin

**Affiliations:** ^1^ Department of Advanced Materials Engineering Kyonggi University Gyeonggi Republic of Korea; ^2^ School of Chemical and Biological Engineering Institute of Chemical Processes Seoul National University Seoul Republic of Korea; ^3^ Department of Energy and Materials Engineering Dongguk University‐Seoul Seoul Republic of Korea; ^4^ Department of Materials Science and Engineering Korea University Seoul Republic of Korea

**Keywords:** active learning, alloy catalyst, density functional theory, oxygen evolution reaction, water electrolysis

## Abstract

The development of efficient electrocatalysts for the oxygen evolution reaction (OER) remains a challenge in alkaline water electrolysis. Discovering optimal compositions for complex multi‐element electrocatalysts is time‐intensive and resource‐consuming due to the vast compositional space. Here, we propose a hybrid computational strategy that integrates active learning (AL)‐assisted screening with density functional theory (DFT) calculations to accelerate the development of multielement alloy electrocatalysts. Our approach employs 15 cycles of active learning, with 20 NiCoFe (NCF) surface configurations evaluated per cycle, totaling 300 DFT calculations. To minimize computational cost, we utilized scaling relationships and calculated only *O and *OH adsorption energies, while the AL featurization incorporated surface configuration information. This methodology filtered the compositional space of Ni_x_Co_y_Fe_100‐x‐y_ alloys and identified the optimal composition of Ni_55_Co_10_Fe_35_. Experimental validation demonstrated that the optimized NCF exhibits accelerated OER kinetics and practical activity, as evidenced by a low Tafel slope of 43.07 mV dec^−1^ while maintaining 98.68% of its initial performance over 24 h at 10 mA cm^−2^. This study demonstrates the effectiveness of the hybrid AL‐DFT strategy for resource‐efficient composition screening within the NiCoFe ternary alloy system and highlights its potential for application to other multicomponent catalyst systems following appropriate system‐specific model development and validation.

## Graphical abstract

A surface‐informed active learning framework integrated with density functional theory enables resource efficient screening of the NiCoFe ternary composition space. Experimental validation identifies Ni_55_Co_10_Fe_35_ as the optimal composition, exhibiting favorable oxygen evolution reaction kinetics. This approach demonstrates the potential of data‐driven computational screening for accelerating multicomponent electrocatalyst discovery.

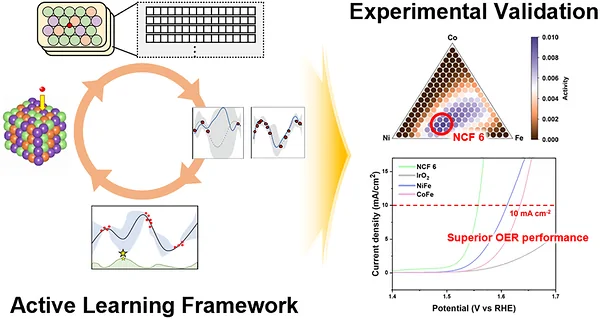

## 1 Introduction

The increased use of fossil fuels has led to a significant rise in environmental pollution, primarily driven by the rapid increase of greenhouse gas emissions [1, 2, 3]. Renewable energy resources have emerged as a carbon‐free and sustainable alternative; however, their intermittent nature, dependent on weather conditions, poses challenges for maintaining a stable energy supply [4, 5, 6]. To address this issue, alkaline water electrolysis has shown promise for hydrogen production owing to its stability and efficiency in utilizing renewable energy [7]. Nevertheless, it faces critical challenges such as sluggish oxygen evolution reaction (OER) kinetics and high overpotentials, which significantly limit OER efficiency [8, 9, 10]. While OER catalysts play a critical role in enhancing efficiency, the high cost and poor stability of noble metal catalysts have driven the urgent search for earth‐abundant alternatives [11, 12].

Transition metal‐based catalysts have been developed to enhance OER activities owing to their high conductivity, abundant valence states, and large surface areas, enabling superior catalytic performance [13, 14, 15]. Their catalytic active centers are generally transition metal atoms because the d orbitals of elements with underfilled electrons can accept single electrons or electron pairs. Subsequently, the acceptor and donor can form intermediates, thereby reducing the activation energy of the reaction and promoting the reaction at lower energy for effective water splitting [16, 17]. This fundamental understanding has highlighted that d‐band center engineering through the combination of various metal elements becomes essential to increase the intrinsic activity of alloy electrocatalysts [18, 19]. Ni–Fe and Ni–Co alloys represent typical examples where the OER kinetics are adjusted by modulating the d‐band center with solid solution structures of different metal elements for water splitting [20, 21]. Building upon this principle, the intrinsic activity of electrocatalysts for water splitting can be further improved by combining multiple metal elements to form highly efficient alloy‐based electrocatalysts [22, 23, 24].

Despite these advances, many studies have been conducted to identify optimal alloy composition through empirical approaches, yet a systematic strategy for alloy design remains lacking. Moreover, experimentally screening all possible compositions of alloy‐based electrocatalysts is nearly impossible owing to the vast parameter space of alloy compositions. The challenge becomes even more complex when considering ternary or higher‐order systems, where the number of possible configurations increases exponentially as the number of elements increases. Therefore, it is critical to initiate research aimed at establishing a systematic framework for the development of alloy‐based electrocatalysts, moving away from the conventional trial‐and‐error approaches toward more rational design strategies.

The recent development of active learning (AL) has motivated its application as a promising material design tool for electrocatalysts because of its time and cost efficiency [25, 26, 27]. To elucidate the intrinsic relationships between descriptors and catalytic activity, AL has been employed to screen catalysts that exhibit high electrocatalytic performance. For example, AL‐driven material design of alloy catalysts for water electrolysis has demonstrated the capability to provide optimal compositions, using Gaussian process regressor (GPR) models to identify compositions with high catalytic activity [28, 29, 30]. Additionally, AL approaches have been used to predict OER and hydrogen evolution reaction performance based on the atomic‐scale properties of single‐atom catalysts [31, 32, 33]. These studies have shown remarkable success in predicting catalyst performance and accelerating the discovery process.

However, existing AL studies often rely predominantly on the atomic properties of individual metals, such as electronegativity, atomic radius, and d‐band characteristics [34, 35]. While these approaches have shown success for single‐atom catalysts and simple binary systems, such models cannot be readily implemented for complex alloy electrocatalyst systems owing to the intricate nature of atomic compositions and arrangements at catalytic surfaces. The surface configuration of alloy catalysts, including local atomic environments, coordination numbers, and electronic interactions between different metal species, plays a crucial role in determining catalytic activity. This complexity necessitates a more sophisticated approach that can capture the surface structural information of alloy systems. Therefore, a surface configuration information‐based AL algorithm is proposed to accelerate the design and discovery of desirable ternary alloy catalysts.

In this study, we combine density functional theory (DFT) calculations with surface configuration information‐based AL to develop an accelerated screening protocol, followed by experimental verification to optimize the composition of ternary alloy catalysts for enhanced OER performance. As a representative system, we selected the Ni–Co–Fe ternary alloy to evaluate the feasibility of AL‐driven design in optimizing alloy composition to maximize OER activity. One of the primary postulates for selecting this ternary system was that the solid solution of Co, Fe, and Ni could result in an upward shift of the d‐band center of Ni, bringing it closer in energy to the O 2p orbital, thereby reducing the energy difference for the OER process and improving water‐splitting efficiency [36, 37, 38]. This electronic structure modulation through multi‐element alloying represents a promising strategy for enhancing catalytic performance. We developed a comprehensive active learning framework that efficiently samples DFT‐calculated properties of alloy‐based electrocatalysts and correlates the composition of ternary alloys with their catalytic activities. The framework employs 15 cycles of active learning, with 20 NiCoFe surface configurations evaluated per cycle, totaling 300 DFT calculations. To minimize computational cost while maintaining accuracy, we utilized scaling relationships and calculated only *O and *OH adsorption energies, as these intermediates are critical for determining OER activity. The AL featurization incorporated detailed surface configuration information, enabling the model to capture the complex relationships between local atomic environments and catalytic performance.

Following the computational screening and optimization, NiCoFe ternary alloys for OER electrocatalysts were synthesized to validate the AL‐based predictions of electrocatalytic properties. The experimental validation confirmed the effectiveness of our approach, with the optimized NiCoFe alloy (Ni_55_Co_10_Fe_35_) demonstrating superior performance compared to conventional noble metal‐based catalysts and binary alloy catalysts. The optimal composition achieved remarkable electrochemical performance metrics, including a Tafel slope of 43.07 mV dec^−1^, an overpotential of 327 mV at a current density of 10 mA cm^−2^, and a charge transfer resistance of 27.84 Ω. These results establish the effectiveness of the hybrid AL‐DFT framework for resource‐efficient composition screening within the NiCoFe ternary alloy system and provide a foundation for evaluating its potential application to other multicomponent catalyst systems.

The success of this approach paves the way for systematic catalyst discovery and represents a significant step toward sustainable and efficient water electrolysis technologies.

## 2 Results and Discussion

### 2.1 Active Learning Framework

Previous studies have demonstrated the OER mechanism in alkaline conditions [4, 39]. The reaction pathway under alkaline conditions typically consists of four cooperative steps of proton‐coupled electron transfer: (1) H_2_O molecules converted to *OH, (2) *OH transforming to *O, (3) *OOH generated through the coupling of H_2_O and *O through proton‐electron transfer, and (4) O_2_ gas released by deprotonating *OOH. Based on the binding energies of the respective intermediates, each elementary step has a unique free energy, and the rate‐determining step is identified as the step with the largest free energy difference ($G=\;\max[\Delta G_{1},\Delta G_{2},\Delta G_{3},\Delta G_{4}]$), which also defines the theoretical overpotential of the whole reaction. For a catalyst to show ideal activity, all four steps should require the same magnitude of reaction free energy. However, this ideal behavior is prevented by the linear correlation between the adsorption energies of the intermediates. It has been established that *OH and *OOH primarily favor identical binding sites, with each sharing a single bond between the oxygen atom and the active site. Consequently, the interaction between the intermediates and the catalyst surface is the only factor that determines the binding energies of *OH and *OOH, which show a linear relationship. As a result, the value of Δ*G*_OOH_ can be directly calculated from Δ*G*_OH_, and the adsorption energies of the corresponding species are independent of the *O binding energy. This scaling relationship implies that accurate prediction of OER activity requires determining both *O and *OH adsorption energies independently.

For ternary alloy systems, determining these adsorption energies becomes particularly challenging because their elements are randomly distributed throughout the structure, creating diverse local environments at potential active sites. The varied elemental arrangements result in multiple unique active sites within a single alloy particle, meaning that the overall catalytic activity can only be accurately determined by evaluating adsorption energies across all these possible binding configurations. The combinatorial nature of this problem, which requires extensive sampling of both compositional space and local binding environments, makes exhaustive DFT screening computationally prohibitive. Given these fundamental challenges, we developed a surface configuration information‐based AL model to predict the OER activity across the entire compositional space. A GPR model was selected to establish the connection between computed DFT energies and local atomic environments surrounding reaction intermediates at catalytic surface. GPR offers several advantages over other regression techniques, including the ability to make reliable predictions near training data points without assumptions about the underlying functional form, making it particularly well‐suited for predicting OER activity in alloy systems that lack a well‐defined theoretical relationship. Additionally, GPR provides quantitative uncertainty estimates at each prediction point, enabling the identification of candidates that require further calculations to enhance model accuracy. To ensure the robustness of our modeling approach, GPR performance was compared against seven established active learning algorithms including linear regression, k‐nearest neighbors, support vector regression, random forest regression, gradient boosting regression, AdaBoost regression, and bagging regression (Figures S1 and S2). GPR consistently outperformed alternative methods, demonstrating superior predictive accuracy while providing essential uncertainty quantification for the acquisition function. Thus, the GPR model was used to predict the *O and *OH adsorption energies across the entire range of ternary alloy adsorption sites.

The integrated AL/DFT workflow adopted in this study is illustrated in Figure 1. The process begins with the generation of a NiCoFe ternary alloy dataspace, from which an initial training dataset is randomly selected. The core of our approach employs an active learning framework that iteratively refines catalyst predictions through cycles of DFT calculations, model training, and candidate sampling. This iterative process continues until convergence criteria are met, ultimately identifying optimal alloy compositions for experimental validation. The active learning process advances through the following steps: data augmentation, model training, and catalytic activity prediction. Feature engineering, which involves crafting a concise yet informative set of features, is a crucial step in constructing AL models. The selection of input features significantly influences a model's accuracy. Given the complex nature of ternary alloys, it is essential to select features that are both time‐efficient and capable of comprehensively depicting the atomic structures of active sites while presenting interactions between the surface and adsorbate. Therefore, the local atomic environment of the adsorbate is represented by dividing the surrounding atoms into groups based on their distance from the adsorbate, assuming that atoms within the same group contribute equally [40]. This method efficiently represents the complex multi‐elemental motifs. Figure 2a illustrates the featurization process of the *O‐adsorbed slab, which consists of Ni, Co, and Fe. The AL approach was used to establish a nonlinear regression relationship between the atomic parameters and adsorption energies of *O and *OH. Both *O and *OH were adsorbed at the face‐centered cubic (FCC) hollow site, as DFT calculations on pure Ni, Fe, and Co metal surfaces identified this site as the most favorable (Tables S2 and S3). Two separate models were created to capture the different binding characteristics of the two adsorbates, each predicting the *O and *OH binding energies for the given surface motif.

**FIGURE 1:**
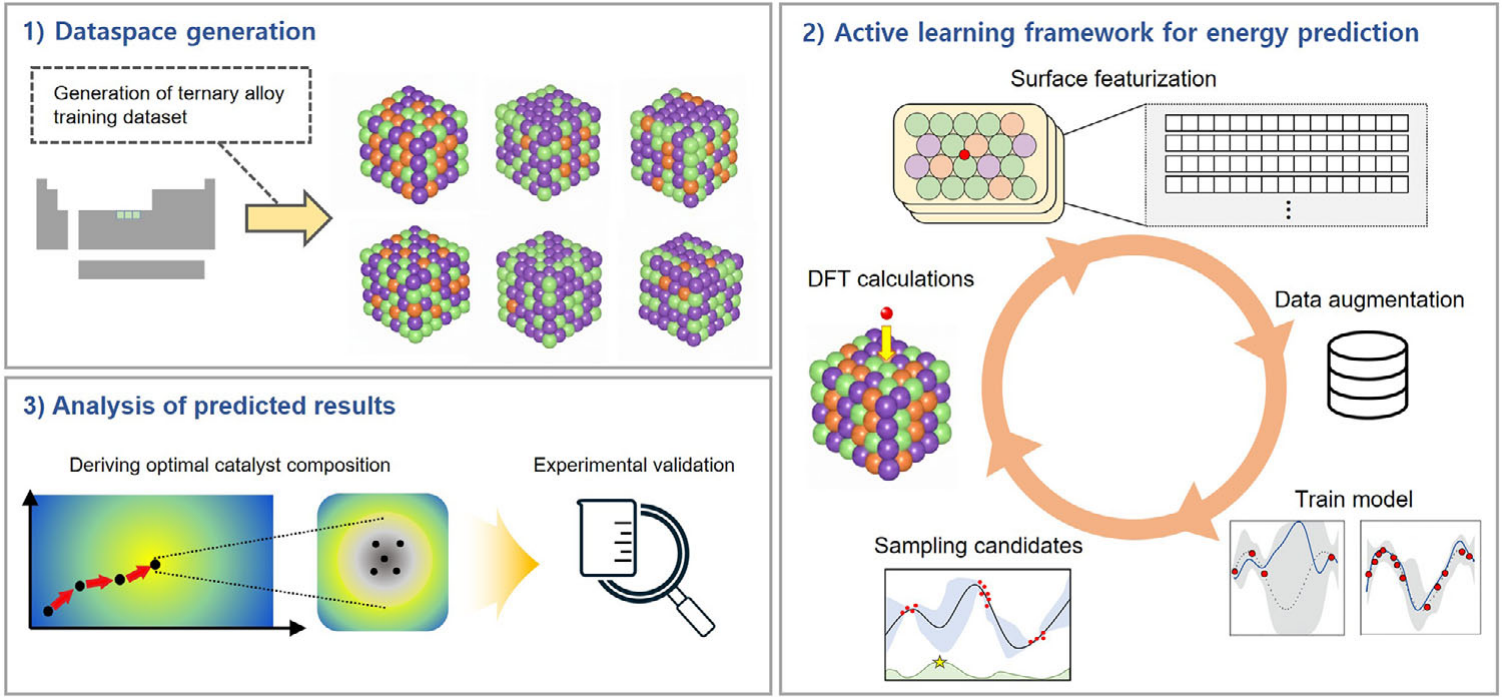
Integrated AL/DFT workflow for ternary alloy OER catalyst discovery. The workflow comprises three key stages: (1) generation of the NiCoFe ternary alloy dataspace and random selection of the initial training dataset; (2) active learning framework for adsorption energy prediction using GPR, including DFT calculations, surface featurization, model training, and iterative candidate sampling; (3) analysis of predicted results to derive optimal catalyst compositions and experimental validation of OER performance.

**FIGURE 2:**
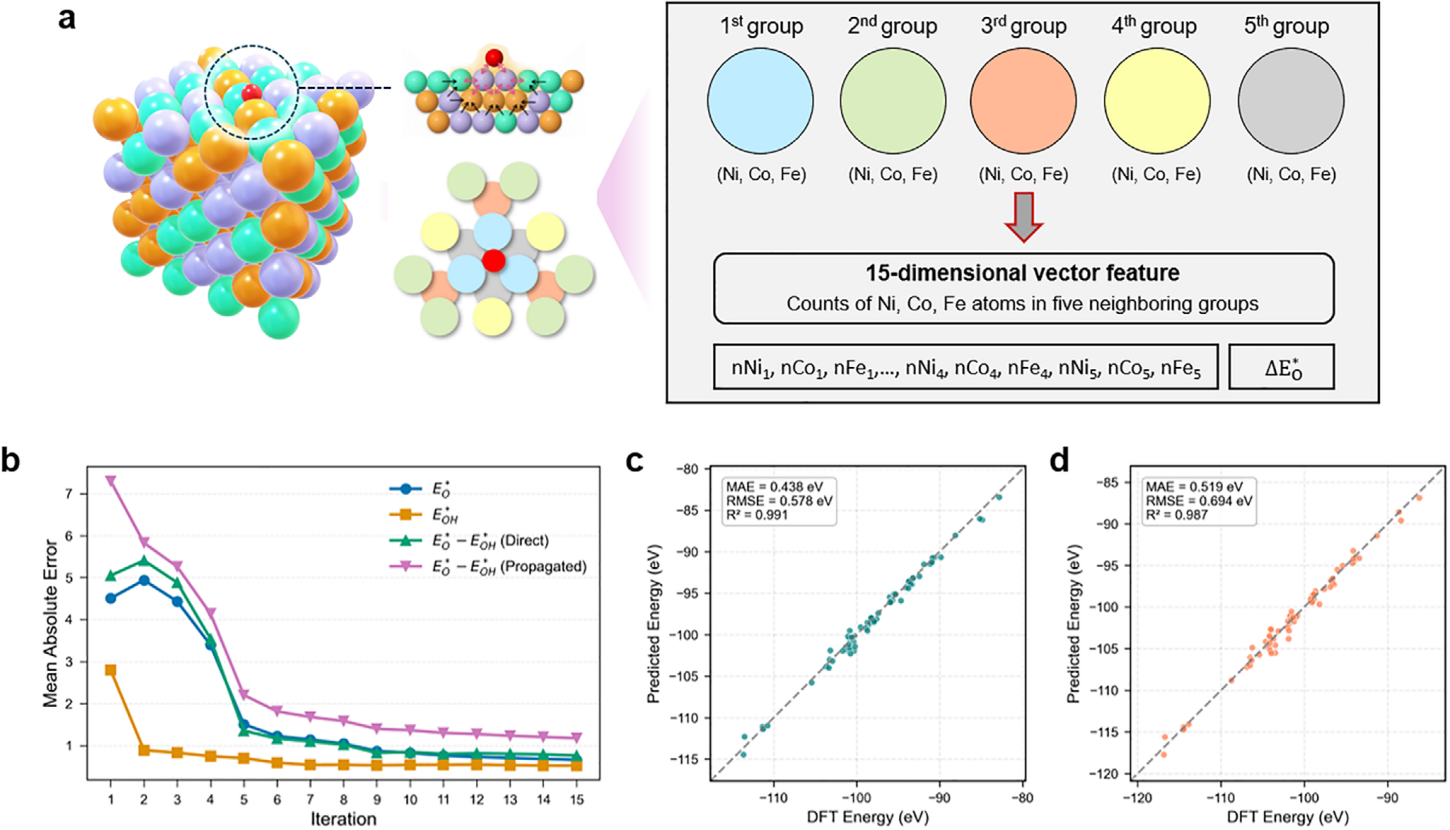
Active learning framework and model performance for OER descriptor prediction. (a) Featurization scheme for the O‐adsorbed NiCoFe ternary alloy surface. Atoms surrounding the adsorbate are divided into five neighboring groups based on distance, with each group characterized by the counts of Ni, Co, and Fe atoms, forming a 15D feature vector along with the oxygen adsorption energy. (b) Mean absolute errors during the active learning iterations. Four metrics are tracked: mean absolute errors for O adsorption energy ($E_{O}^{*}$) and OH adsorption energy ($E_{\mathrm{OH}}^{*}$), the direct prediction error for the OER descriptor ($E_{O}^{*}\;E_{\mathrm{OH}}^{*}$), and the propagated error estimated from individual adsorption energy uncertainties. (c, d) Parity plots showing predicted versus DFT‐calculated energies for (c) *O adsorption and (d) *OH adsorption on the test set, respectively.

To reduce the computational efforts in the DFT calculations required for developing data for a large number of combinations, we employed an acquisition function to navigate the data sampling process. Expected improvement (EI) is one of the most common acquisition functions, balancing exploration and exploitation by calculating the expected improvement each point offers over the current best predictive mean [41]. Considering the error characteristics of predictions, we propose using the acquisition function to navigate the vast search space. By converting the current best mean to a fixed optimal adsorption energy value, the algorithm is guided to search the optimal space more effectively, improving the accuracy in identifying promising catalysts. Figure S3 outlines the full algorithm of the acquisition function. The optimal value is chosen based on the descriptor/activity relationship derived from the Sabatier principle, which demonstrates that the intermediates exhibit ideal bond strengths. Based on the volcano relationship between the overpotential and Δ*G*_O_ − Δ*G*_OH_, the optimal value is set to 1.6 eV, corresponding to a 5.3 eV difference between the adsorption energies of the two intermediates. Detailed calculations of the optimal adsorption energy are provided in the Supporting Information.

The EI acquisition function advises selecting the subsequent sampling points where both the uncertainty and the potential to become the new optimal value are high. This is achieved by searching for surfaces with near‐optimal adsorption energies and large uncertainty. At each iteration of the learning cycle, 20 surfaces with the largest average EI values obtained from the *O and *OH models are selected for further DFT calculations. Replaying the procedure over the completed dataset with batch sizes from 1 to 40 changed the descriptor error at a fixed budget of 100 DFT calculations by less than 0.01 eV (Figure S4), and configurations selected within the same batch were found to carry largely independent information, with the median fraction of predictive variance shared with earlier selections remaining near 0.15 across the batch (Figure S5). The batch size of 20 was therefore chosen for computational throughput, allowing 40 slab relaxations per cycle to be run concurrently. The data augmentation process was iteratively performed until the adsorption energy prediction errors converged, as shown in Figure 2b and Figure S6. Across 15 cycles, 300 configurations were selected. During geometry optimization, 46 of these showed significant shifts in the *O or *OH adsorbate position from the initial FCC hollow site. Since the model requires comparable adsorption sites for both O and OH on the same surface, these configurations were excluded, leaving 254 configurations, divided into 190 training and 64 test surfaces. This ensures that all data points in the analysis represent surfaces where both adsorbates maintain stable positions at the same site. In addition, 5‐fold cross‐validation was employed at each active learning cycle to optimize hyperparameters as the training set expanded (Table S4). This dynamic optimization strategy prevented overfitting while maintain predictive accuracy throughout the learning process.

### 2.2 Model Performance and Validation

The mean absolute error (MAE) saturation after 15 active learning cycles indicates that additional calculations would yield minimal model improvement. The learning curve analysis in Figure 2b reveals characteristic two‐phase behavior with an initial rapid improvement phase (cycles 1–5) where MAE decreased dramatically from 4.51 to 1.51 eV for $E_{O}^{*}$ predictions and from 2.80 to 0.70 eV for $E_{\mathrm{OH}}^{*}$ predictions, followed by a gradual refinement phase (cycles 6–15) with diminishing returns where MAE further improved to 0.66 and 0.52 eV, respectively. For the target descriptor ($E_{O}^{*}\;E_{\mathrm{OH}}^{*}$), the direct prediction approach achieved a MAE reduction from 5.05 to 0.76 eV over the 15 cycles, demonstrating effective learning. This convergence pattern validates that the active learning approach efficiently explores the compositional space without excessive computational cost, achieving > 85% error reduction in the initial five cycles and > 90% overall improvement. The final GPR model performance after 15 active learning cycles, illustrated in Figure 2c,d, demonstrates remarkable accuracy for both adsorbates with MAE values of 0.438 eV for $E_{O}^{*}$ and 0.519 eV for $E_{\mathrm{OH}}^{*}$ across 75 test surface configurations. The corresponding RMSE values of 0.578 eV and 0.694 eV, along with high *R* ^2^ values of 0.991 and 0.987 for $E_{O}^{*}$ and $E_{\mathrm{OH}}^{*}$ respectively, indicate excellent predictive capability with minimal systematic bias. These low error values validate that our configuration‐based active learning framework effectively captures the complex adsorption mechanisms on multi‐metallic surfaces with only 190 cumulative training samples. A detailed methodology for the active learning process is provided in the Methods section, with additional computational details available in the Supporting Information.

Applying the converged GPR model to all 280 000 candidate surface configurations yields a broad, right‐skewed distribution of predicted Δ*G*_O_ − Δ*G*_OH_ values (Figure S7), with a median of 0.43 eV and an interquartile range of 2.24 eV. 72% of configurations fall below the optimal value of 1.6 eV, confirming that NiCoFe terminations predominantly bind oxygen intermediates too strongly, and only 2.5% lie within ±0.1 eV of the optimum. Near‐optimal configurations thus constitute a small and irregularly distributed subset of the compositional space. The governing challenge is therefore not to find an acceptable configuration but to identify the best one, which motivates a guided search strategy.

To assess the data efficiency of the framework, we retrospectively evaluated model accuracy as a function of training‐set size against a fixed test set of 59 surface configurations withheld before any model was trained. The descriptor error converges after ≈80 DFT calculations to a MAE of 0.028 eV (Figure S8a), so converged accuracy is attained from ≈0.03% of the 280 000 candidate configurations. For reference, a constant predictor returning the training‐set mean gives a MAE of 0.128 eV, so the model removes 79% of the error of this trivial baseline. Repeating the analysis with 30 independent random initializations of the 20‐point seed set gives a final MAE of 0.026 eV that varies by less than 0.001 eV between repeats, with convergence reached between 20 and 120 calculations, confirming that the outcome does not depend on the initial training data (Figure S8b). Comparing the EI acquisition with random selection at matched budget shows the trade‐off characteristic of an acquisition function. Global test error is comparable between the two (Figure S8c), whereas EI locates the configuration with the most favorable descriptor in the candidate pool after 80 calculations, which random selection does not achieve within the 180 calculations examined (Figure S8d), and EI is more accurate on the 10% of test configurations with the largest descriptor values from the same point onward (Figure S9). We note that this comparison is conservative, as the candidate pool consists of configurations selected by EI during the original campaign. The acquisition function therefore preferentially resolves the near‐optimal region rather than the compositional space uniformly, which is the behavior relevant to catalyst discovery.

The strategic effectiveness of the acquisition function is demonstrated in Figure S10, which shows histograms of DFT‐calculated values for the 254 configurations selected during active learning. The *O binding energies span ‐0.859 to 0.444 eV, while *OH energies range from ‐2.115 to 0.679 eV. The relatively narrow distributions indicate focused exploration around catalytically relevant binding energies, demonstrating the efficiency of the expected improvement acquisition function in identifying informative surface configurations while avoiding redundant calculations.

### 2.3 Composition Optimization from AL Model

To apply AL for optimizing the Ni–Co–Fe composition, three boundaries were established. NiCoFe alloys with an FCC structure were selected for computational assessment and experimental verification. The FCC structure of the alloys offers several advantages for catalytic performance, primarily owing to the atomic arrangement and the resulting surface properties. For instance, the FCC structure has a high surface area owing to its dense atomic packing, and the low‐index facets, such as {111}, provide favorable adsorption sites, exhibiting high catalytic activity. Additionally, the FCC structure provides stable coordination environments for surface atoms, resulting in enhanced stability for catalytic applications [42, 43, 44]. We also considered the variation of alloy compositions with 5% intervals in the AL framework for experimental validation. The activity of the alloy catalyst is computed by multiplying the probability associated with each binding site with the metric representing the calculated $E_{O}^{*}-E_{\mathrm{OH}}^{*}$ relative to the optimal value. This calculation accounts for the likelihood of the surface motif at each composition of the ternary alloy. The Boltzmann distribution is utilized as the probability measure of the reaction (see Supporting Information for further details) [45, 46].

Figure 3a shows the final catalytic activity diagram of the NiCoFe ternary system. A noticeable maximum activity of 0.00966 is observed at the composition Ni_55_Co_10_Fe_35_. A gradient is evident where the activity gradually decreases as the Ni ratio decreases. The locally optimal compositions using a 5 at% grid search on the composition space are presented in Table S5. These optimal compositions predominantly consist of Ni and Fe, with minimal Co content. As shown in Table S6, calculations on pure metal surfaces reveal that Ni exhibits $E_{O}^{*}-E_{\mathrm{OH}}^{*}$ value of 3.37 eV, which is closest to the optimal value of 5.3 eV compared to other metal surfaces. This suggests that Ni plays a crucial role in enhancing catalytic activity, while the other metals primarily act as supports. Moreover, the activity distribution of the multi‐metallic alloy reveals a nonlinear variation within the ternary system, demonstrating the “cocktail effect” [47, 48]. This effect refers to the synergistic interactions among the composing elements, leading to properties that exceed or deviate from those predicted by the mixture rule. The cocktail effect results in either enhanced or unexpected properties owing to interstitial strengthening and intermetallic precipitation strengthening. Overall, the combination of elements can produce enhanced or unexpected properties, which are evident in multi‐metallic alloy systems.

**FIGURE 3:**
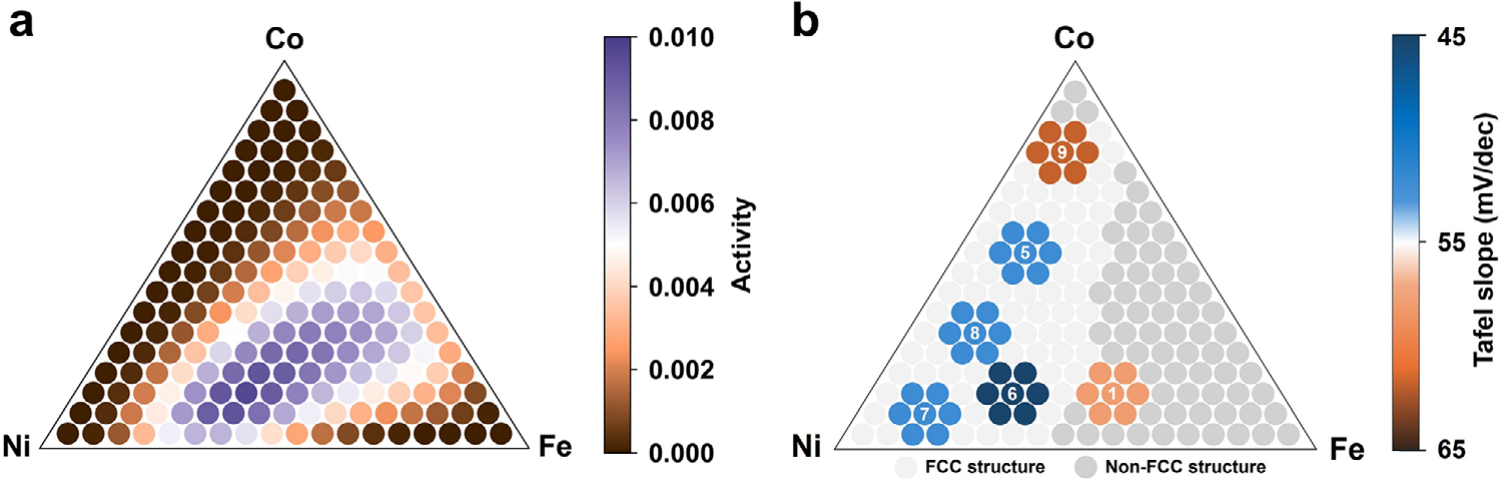
Comparison between the active learning predicted OER activity and the experimentally obtained Tafel slope map of the NiCoFe ternary system. (a) Ternary activity map of the NiCoFe system, with the purple region indicating optimal catalyst composition predicted by the AL model. (b) Tafel slope map of the NiCoFe ternary system, compared with active learning predicted trends to validate the compositional dependence of OER kinetics.

Figure S11 illustrates the composition‐weighted energy distribution for the optimal NiCoFe alloy, highlighting the critical importance of considering surface motif probabilities in catalytic activity predictions. The distribution reveals that the optimal Ni_55_Co_10_Fe_35_ composition exhibits a high probability for surface motifs with near‐optimal $E_{O}^{*}-E_{\mathrm{OH}}^{*}$ values, providing a quantitative explanation for the enhanced catalytic activity observed at this composition. The composition‐weighted mean of 5.500 eV is close to the ideal value of 5.3 eV, confirming that the most probable surface configurations in this alloy are those with favorable binding characteristics. The 0.673 eV difference between weighted and unweighted means demonstrates that the statistically dominant surface motifs in the optimal composition cluster around energetically favorable binding regimes, while rare surface configurations with extreme binding energies have minimal practical impact on overall performance.

The catalytic activities of the samples were experimentally evaluated to assess the AL‐driven composition screening (Figure 3b). NCF6 (Ni_55_Co_10_Fe_35_), identified as the optimal composition by the AL‐DFT framework, exhibits the lowest Tafel slope of 43.07 mV dec^−1^ among the experimentally evaluated compositions (Table S7 and Figure S12). Although the predicted high‐activity region does not completely overlap with the experimentally observed low‐Tafel‐slope region, the identification of Ni_55_Co_10_Fe_35_ as the experimentally most favorable composition supports the utility of the AL‐DFT framework for narrowing the NiCoFe composition space. The difference between the two activity landscapes reflects the distinct quantities represented by the computational and experimental metrics. The AL‐DFT framework evaluates OER activity using a descriptor derived from the adsorption energetics of *O and *OH intermediates over local alloy configurations, whereas the experimental Tafel slope reflects the overall reaction kinetics and can be influenced by additional physicochemical factors not explicitly represented in the present model. Accordingly, the comparison between the predicted and experimental activity landscapes is interpreted as validation of composition screening rather than as a direct quantitative correspondence between the two maps.

### 2.4 Characterization of NiCoFe Alloy

In addition to experimental validation of the highest catalytic activity of NCF6 for the OER process through AL‐driven composition screening, material characterization of NCF6 was conducted to investigate the scientific origin of its enhanced catalytic activity. The crystal structure of NCF6 reveals an FCC structure (JCPDS card#03‐1109). A diffraction peak at 26° indicating amorphous carbon is observed for all compositions, suggesting the formation of amorphous carbon on the alloy nanostructure (Figure S13). Exploring the oxidation states of the elements in NCF6 provides further insights into the chemical structure of the alloy (Figure S14 and Table S8). Ni 2p X‐ray photoelectron spectroscopy (XPS) profiles show peaks at 852.98 and 870.28 eV, corresponding to the metallic state of Ni, confirming the presence of Ni metal. Similarly, Co 2p and Fe 2p spectra revealed metallic states, identified by peaks at 777.8 and 792.8 eV for Co 2p and 707.2 and 720.3 eV for Fe 2p. The metallic states of Ni, Co, and Fe confirm the formation of alloy particles. Deconvoluted peaks relating to metal‐oxygen bonding were also observed, with peaks at 854.94, 856.08, 872.24, and 873.39 eV in the Ni 2p spectra; 779.98, 781, 794.98, and 796 eV in the Co 2p spectra; and 710.1, 711.5, 723.2, and 724.6 eV in the Fe 2p spectra. To further clarify the origin of the surface oxygen species, the C 1s and O 1s spectra of pristine NCF6 were analyzed (Figure S15). The C 1s spectrum is dominated by the C–C/C–H component at 284.6 eV, with a higher‐binding‐energy contribution associated with oxygen‐containing/defect‐related carbon species. The O 1s spectrum was deconvoluted into components centered at 530.1, 531.4, and 533.2 eV, which are assigned to metal–oxygen species, hydroxyl‐related oxygen species, and adsorbed/carbon‐related oxygen species, respectively. Survey XPS analysis gives an O/(Ni + Co + Fe) atomic ratio of 4.38 for pristine NCF6. The presence of multiple oxygen‐containing species indicates that the measured oxygen signal cannot be attributed exclusively to oxidation of the constituent metals. Owing to the surface‐sensitive nature of XPS, the O/(Ni + Co + Fe) ratio therefore reflects the overall near‐surface oxygen content rather than the stoichiometric extent of metal oxidation [49, 50]. A direct quantitative comparison of the O/(Ni + Co + Fe) ratio before and after OER was not performed because recovery of the catalyst from the Nafion‐containing ink on the glassy carbon electrode would not provide directly equivalent specimens for quantitative XPS analysis.

The morphology of NCF6 reveals spherical alloy nanoparticles surrounded by a carbon shell, with a lattice fringe of 0.22 nm corresponding to the {111} plane of the FCC structure (Figure S16). Scanning transmission electron microscopy‐energy dispersive X‐ray spectroscopy (STEM‐EDS) mapping confirms the homogeneous distribution of Ni, Co, and Fe within the alloy nanoparticles, demonstrating that the alloy exists as a ternary alloy (Figure 4a). Additionally, oxygen is dispersed within the amorphous carbon. The EDS quantitative analysis indicated that the synthesized composition was consistent with the designed composition. Additionally, oxygen is dispersed within the amorphous carbon (Figure 4b).

**FIGURE 4:**
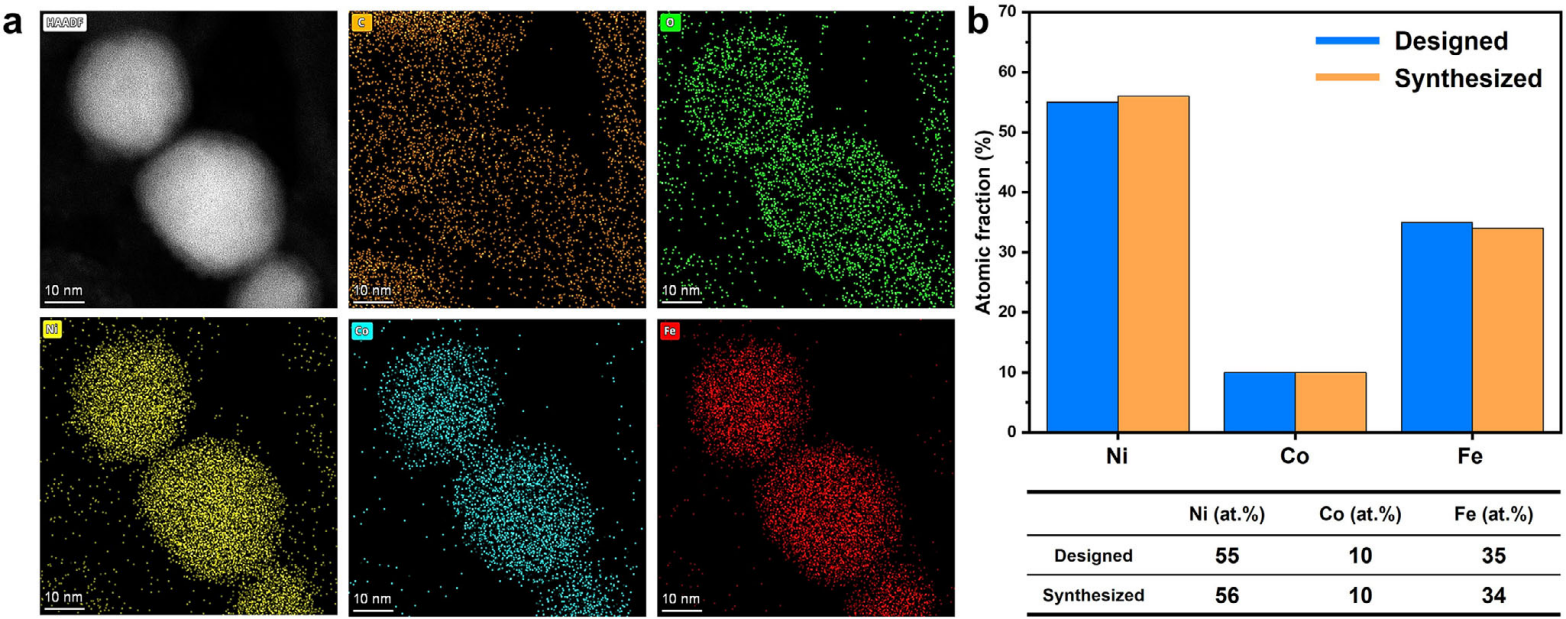
Surface analysis for confirming the formation of alloy in NCF6. (a) HADDF‐ Scanning transmission electron microscopy (STEM) image and corresponding elemental mapping images of C, O, Ni, Co, and Fe in NCF6. (b) Atomic ratio comparison of NCF6 before and after synthesis.

### 2.5 Electrochemical Measurement

The electrochemical OER performance of NCF6 under alkaline conditions was investigated and compared with that of precious metal oxides, binary alloys, and previously reported catalysts (Figure 5 and Table S9). The overpotentials of NCF6, IrO_2_, NiFe and CoFe at 10 mA cm^−2^ were measured to be 327, 523, 380, and 404 mV, respectively (Figure 5a). This result indicates that NCF6 exhibits a lower overpotential than precious metal oxides and binary alloys, representing higher oxygen production efficiency. Additionally, NCF6 exhibits the lowest Tafel slope (43.07 mV dec^−1^) among the catalysts, demonstrating the highest catalytic activity and improved kinetics in the OER process (Figure 5b). The NiCoFe catalyst exhibits higher Ni^3^^+^/Ni^2^^+^ and Fe^3^^+^/Fe^2^^+^ ratios than NiFe, indicating a more pre‐oxidized surface state. (Figure S17 and Tables S8 and S10) As OER involves the oxidation of metal centers into active oxyhydroxide species, such a preconditioned surface state may facilitate the redox transition required for catalytic activation [51, 52]. The charge transfer resistance (*R*_ct_) at the catalyst interface was examined using the semi‐circle diameter in Nyquist plots (*Z*' vs −*Z*'') (Figure 5c). *R*_ct_ for IrO_2_, NiFe and CoFe were 236.36, 71.46, and 98.36 Ω, respectively. NCF6 exhibits the lowest *R*_ct_ (27.84 Ω), implying efficient electron transfer at the catalyst interface, which enhances the efficiency of the OER.

**FIGURE 5:**
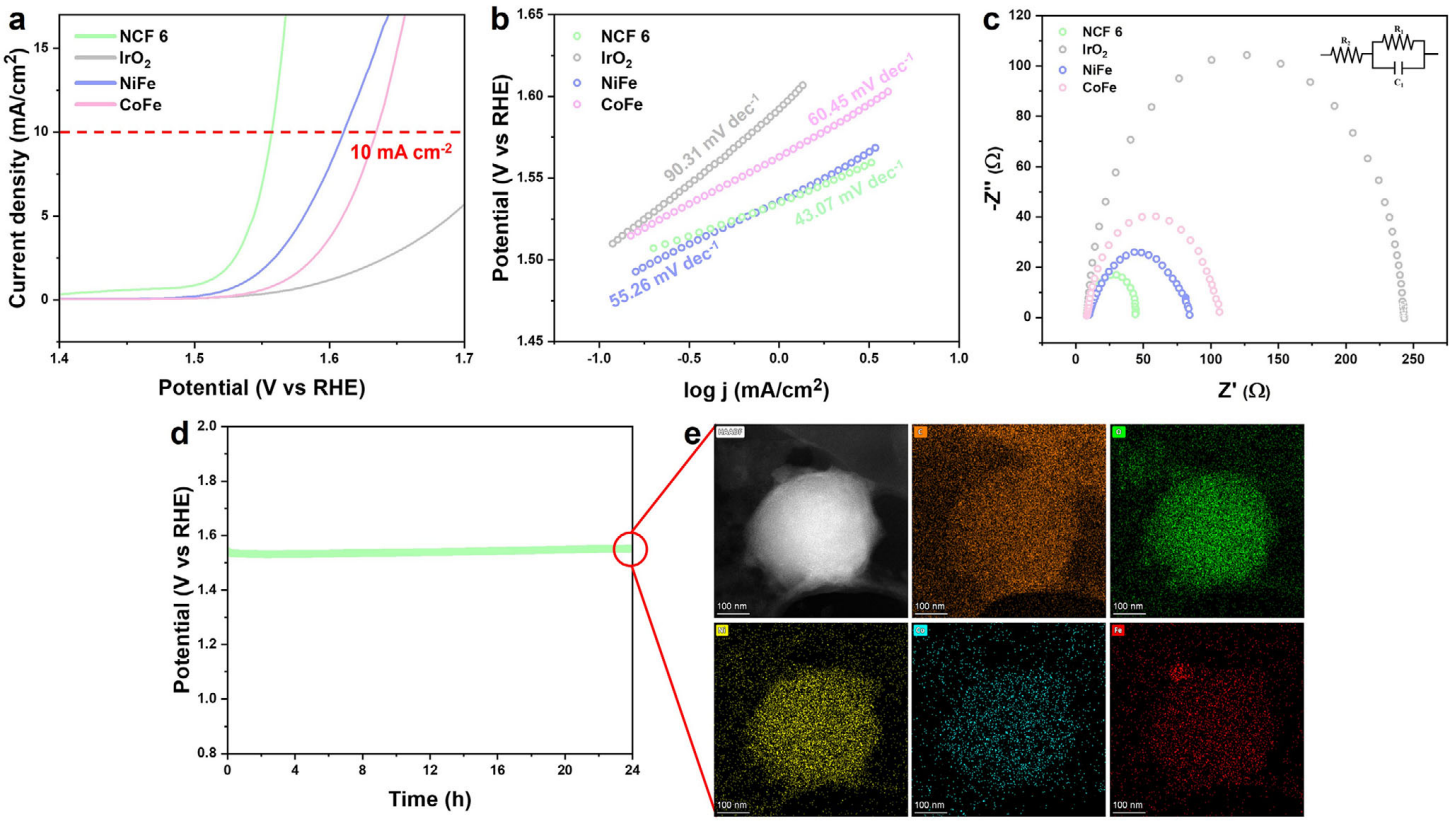
Electrochemical performance evaluation. (a, b) OER polarization curves (scan rate of 5 mV s^−1^) and corresponding Tafel plots for NCF6 (green), commercial IrO_2_ (gray), NiFe (blue), and Co‐Fe (pink) catalyst loaded on rotating disk electrode (2000 rpm) in 1.0 m KOH solution. (c) Nyquist plot (*Z*' vs −*Z*'') of NCF6 (green), commercial IrO_2_ (gray), NiFe (blue), and CoFe (pink). (d) Chronopotentiometry curve of NCF6 under an applied current of 10 mA cm^−2^ (e) Elemental mapping image for C, O, Ni, Co and Fe in NCF6 after the CP test.

The chronopotentiometry test was conducted to evaluate the stability of NCF6, which exhibited an almost constant potential for ≈24 h at 10 mA cm^−2^ (Figure 5d). Following the stability test, STEM‐EDS elemental mapping was performed to examine the elemental distribution of NCF6 (Figure 5e). Ni, Co, and Fe remained distributed throughout the analyzed region without pronounced elemental segregation within the spatial resolution of STEM‐EDS. Together, the stable electrochemical response and post‐OER elemental distribution demonstrate the electrochemical and compositional stability of NCF6 under the tested OER conditions [53, 54].

In the OER mechanisms of water electrolysis, there are primarily two processes: the adsorbate evolution mechanism (AEM), where OH^−^ adsorbs onto the active site to facilitate the reaction, and the lattice oxygen mechanism (LOM), where lattice oxygen directly participates in the reaction [55, 56]. To elucidate the OER mechanism of the Ni–Co–Fe ternary catalyst, pH dependence and catalytic activities in tetramethylammonium hydroxide (TMAOH) were evaluated. IrO_2_ electrocatalysts, which typically follow the LOM, were used for comparison to evaluate their catalytic activity against the Ni–Co–Fe ternary alloy (Figure 6a). IrO_2_ exhibits Tafel slopes of 62.3, 51.88, 88.26, and 164.56 mV dec^−1^ at pH 14, 13.7, 13.5, and 14 (TMAOH), respectively (Figure 6b). For IrO_2_, LOM is likely to govern the OER mechanisms, as indicated by the diminished catalytic activity with decreasing pH. The reduced catalytic activity in TMAOH is attributed to strong electrostatic interactions between TMA^+^ and oxygen‐containing species, which hinder the OER by competing with oxygen evolution [57, 58]. On the other hand, the catalytic activity of NCF6 decreases less compared to that of IrO_2_ in TMAOH (Figure 6c,d). Notably, the absence of significant catalytic activity changes with pH suggests that NCF6 follows the AEM.

**FIGURE 6:**
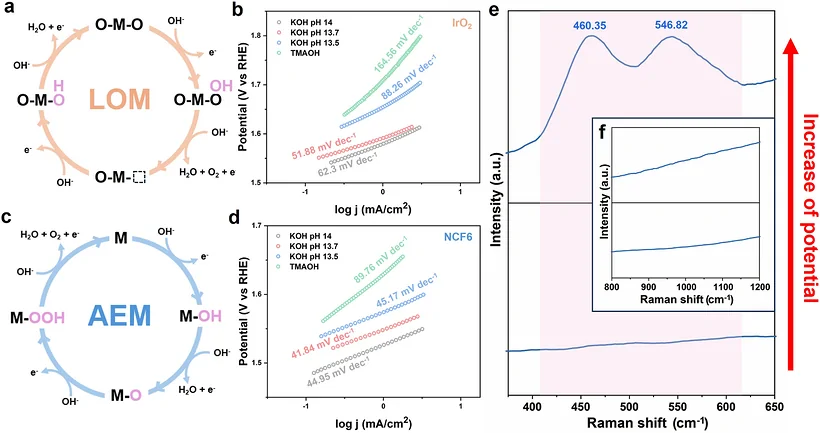
Catalytic activity evaluation and operando Raman spectroscopy for elucidating the OER mechanism. (a) Electron pathway of lattice oxygen mechanism in OER in an alkaline solution. (b) Tafel plots for IrO_2_ in electrolyte of KOH pH 14 (gray), KOH pH 13.7 (pink), KOH pH 13.5 (blue), and tetramethylammonium hydroxide (TMAOH) (pH 14) (green). (c) Electron pathway of adsorbate evolution mechanism in OER in an alkaline solution. (d) Tafel plots for NCF6 in electrolyte of KOH pH 14 (gray), KOH pH 13.7 (pink), KOH pH 13.5 (blue), and TMAOH (pH 14) (green). (e, f) Operando Raman spectra of NCF6 collected under applied potentials (vs Ag/AgCl) after holding each potential for 10 min.

To further identify the active sites responsible for the OER to desired AEM pathway, Raman spectroscopy was employed to monitor the surface structural evolution of NCF6 under operando conditions. Upon applying anodic potentials relevant to the OER, the gradual emergence and intensification of Raman features in the 460–470 and 540–555 cm^−^^1^ regions were observed, corresponding to bending and stretching modes of Ni‐based (oxy)hydroxide, respectively (Figure 6e) [59, 60, 61]. Notably, no additional features associated with lattice oxygen redox were detected in the 800–1200 cm^−1^, supporting an adsorbate‐mediated OER mechanism rather than lattice oxygen participation (Figure 6f) [58, 62, 63]. This result provides direct spectroscopic evidence that the OER activity of NCF6 originates from metal (oxy)hydroxide active sites, consistent with the AEM‐dominated reaction pathway inferred from electrochemical analysis. The formation of Ni‐based (oxy)hydroxide species under OER conditions indicates that the working catalyst surface differs from the alloy surface represented in the present AL‐DFT calculations. The AL‐DFT framework evaluates the OER‐relevant adsorption energetics of *O and *OH intermediates over local configurations of the NiCoFe alloy surface but does not explicitly describe the dynamic surface reconstruction occurring under operando conditions. Accordingly, the framework should be interpreted as identifying a favorable alloy precursor composition rather than explicitly predicting the fully reconstructed operando active phase. Explicit treatment of the reconstructed (oxy)hydroxide surface therefore remains beyond the scope of the present model.

## 3 Conclusion

We demonstrated the integration of the AL assisted DFT protocol and experimental validation to optimize the composition of ternary alloy electrocatalysts for the OER. Through computational predictions, we identified an optimal composition of Ni_55_Co_10_Fe_35_ in the Ni_x_Co_y_Fe_100‐x‐y_ system, which exhibited favorable OER performance among the experimentally evaluated compositions. This approach significantly reduces the time and resource‐consuming process of screening numerous compositions and accelerates the identification of optimal materials. The alloy‐based electrocatalyst with optimal composition (NCF6) not only exhibited excellent catalytic activity but also maintained its performance over 24 h, highlighting its practical applicability in alkaline water electrolysis. This strategy provides a framework for rationally screening NiCoFe alloy compositions by integrating computational predictions with experimental validation. Two extensions follow directly from this work. First, since the featurization scales as 5n for an n‐element system and the acquisition function is independent of the number of elements, the protocol could in principle be extended to quaternary and higher‐order alloys, requiring only a small set of initial DFT calculations for the additional elements. Second, replacing the element‐count features with per‐shell averages of elemental descriptors, such as electronegativity, atomic radius, and d‐band center, would allow a single trained model to be evaluated on elements absent from its training set, eliminating the need to retrain for each new alloy family. These extensions represent prospective methodological directions rather than experimentally demonstrated capabilities, and further computational and experimental validation in other material systems and reaction mechanisms is required to establish the broader applicability of the framework.

## 4 Methods

### 4.1 DFT Calculation Details

The Vienna ab initio simulation package (VASP) was used for all calculations [64, 65]. The effective potential between the ionic cores and electrons was modeled according to the projected augmented wave (PAW) method [66]. The generalized gradient approximation (GGA) in the form of a revised Perdew‐Burke‐Ernzehof (PBE) functional was used to describe the electron exchange‐correlation interactions [67, 68]. The plane‐wave cutoff energy was set to 450 eV, and a k‐point sampling of 4 × 4 × 1 was tested to achieve convergence. The DFT‐D3 scheme of dispersion correction was chosen to describe the long‐range van der Waals (vdW) interactions [69]. The convergence criteria were set to 10^−5^ eV for total energy and 0.05 eV Å^−1^ for the forces on each atom. 2 × 2 supercell models of ternary FCC (111) facets were built into a slab consisting of four layers, repeated periodically in the z direction with 16 Å of vacuum region to avoid interactions between adjacent units. The lattice constant of each multi‐elemental structure was determined using the ratio of metal element precursors.

The reaction free energy of each species can be expressed as

(1) ΔG=ΔE+ΔZPE−TΔS

Here, Δ*E* is the reaction energy, Δ*ZPE* is the difference in zero‐point energies due to the reaction, *T* is the reaction temperature, and Δ*S* is the entropy change. The entropic and zero‐point energy terms are presented in Table S1, following the electrochemical framework and OER thermochemistry developed by Nørskov et al. [70].

### 4.2 Active Learning Model

GPR was used to process the prediction of adsorption energies and measure uncertainties [71, 72], and these values were inserted into the acquisition function that provided surface features for the next active learning cycle. The 15D vectors were fed as input features, where each element of the vector denotes the number of Ni, Co, and Fe atoms in the five neighboring groups around the adsorbate. The construction of these feature vectors is described in detail in the Supporting Information. To investigate the catalytic performance of the active sites with computational efficiency, a single descriptor of $\Delta G_{O}^{*}-\Delta G_{\mathrm{OH}}^{*}$ was selected as the target variable of the model learning [45, 73].

The GPR model is constructed using the squared‐exponential covariance kernel as follows:

(2) $$k\;\left(x_{1},\;x_{2}\right)=\sigma_{s}^{2}\;\exp\left[-\frac{{\left|x_{1}-x_{2}\right|}^{2}}{2l^{2}}\right]+\sigma_{n}^{2}\delta_{12}$$

Here, σ_*s*_ represents the signal level, while *l* denotes for the length scale. Each element of *l* signifies the extent of variation in adsorption energy along that direction. Given that the input vectors (*x*_1_ and *x*_2_) consisted of discrete integers, adjustments were made to the kernels using a step function to enhance the learning process, a method found in literature [74]. The second term $\sigma_{n}^{2}$ represents the white noise kernel, which prevents overfitting of the training data. The code and final datasets are provided at https://github.com/minhee2043/ActiveLearning_OER.

### 4.3 Material Synthesis

Ni_x_Co_y_Fe_100‐x‐y_ alloy electrocatalysts were synthesized using a sol‐gel method, followed by a carbothermal method. For the sol‐gel process, nickel nitrate hexahydrate (Sigma Aldrich, 97%), cobalt nitrate hexahydrate (Sigma Aldrich, ≥ 98%), iron nitrate nonahydrate (Sigma Aldrich, ≥ 98%), and citric acid (DAEJUNG, 99.5%) were directly used without further purification. The metal nitrate precursor and citric acid were mixed in stoichiometric amounts in 30 mL of deionized water. The mixture was stirred for 30 min at room temperature to enable dissolution, followed by continuous stirring at 120°C until a gel was formed. Subsequently, the gel was dried for 10 h at 120°C in a conventional oven and then heated for 6 h in a tube furnace at 800°C under an Ar/H_2_ (5%) atmosphere.

### 4.4 Characterization

X‐ray diffraction (XRD) was conducted with Cu Kα radiation in the 2*θ* range from 20° to 80° with a scan rate of 0.013° s^−1^ (Empyrean, PANalytical). STEM and TEM were used to observe the morphology of the alloy electrocatalysts and determine the elemental distribution of the samples (Spectra 300, Thermofisher Scientific). XPS was employed to measure the oxidation states and identify the components present in a metallic state (NEXSA, Thermofisher Scientific).

### 4.5 Electrochemical Measurements

Electrochemical characterization was performed in 1 m KOH using a three‐electrode system equipped with a rotating disk electrode (RDE) controlled by a potentiostat (Autolab PGSTAT, Metrohm). The metal nitrate precursors and citric acid were mixed at a molar ratio of 1:3 based on total metal ions to citric acid. The catalyst ink was prepared by ultrasonication with 4 mg of sample, 1 mL of deionized water and ethanol solution (volume ratio 3:1), and 30 µL of Nafion solution (Sigma Aldrich, ≈5 wt%). Next, 5 µL of catalyst ink was drop‐cast on the surface of a glassy carbon RDE with a diameter of 3 mm. The catalyst loading was approximately 0.27 mg cm^−2^. It was dried overnight and subsequently used as the working electrode. The RDE was rotated at 2000 rpm during the electrochemical measurements to remove bubbles generated during the reaction. Ag/AgCl (3.5 m KCl) and a Pt sheet were utilized as the reference electrode and counter electrode, respectively. Linear sweep voltammetry (LSV) was carried out at a scan rate 5 mV s^−1^ from 1.23 to 1.85 V. The potentials were calibrated against a reversible hydrogen electrode, and all polarization curves were iR‐compensated. Electrochemical impedance spectroscopy was conducted over the frequency range of 0.1–100 kHz with a sinusoidal amplitude of 5 mV.

Tafel plots were obtained to evaluate the OER activity using iR‐corrected LSV polarization curves. The Tafel slopes were calculated using the following equation:

(3) $$\eta =a+b\log\left(j\right)$$

Here, *η* is the overpotential, *a* is the constant of Tafel slope equation, and *b* is the Tafel slope. Electrochemical measurements for CP were conducted in a 1 m KOH solution. Raman spectroscopy was employed to analyze the carbon structure and to study the OER mechanism under applied potentials using a 532 nm laser (HEDA‐SERA, NOST). In addition, the OER mechanism was further elucidated by evaluating the catalytic activities in KOH and TMAOH (Sigma‐Aldrich, 1.0 m solution).

## Author Contributions

**Hayun Jeon**: investigation, validation, data curation, visualization, writing – original draft, review & editing. **Minhee Park**: software, methodology, validation, data curation, visualization, writing – original draft, review & editing. **Won Bo Lee**: software, methodology, validation, and writing – review & editing. **Seyoung Park**: investigation, validation, data curation, writing – review & editing. **YongJoo Kim**: conceptualization, supervision, software, funding acquisition, and writing – review & editing. **Sungwook Mhin**: conceptualization, supervision, funding acquisition, and writing – review & editing

## Conflicts of Interest

The authors declare no conflicts of interest.

## Supporting information




**Supporting File**: advs78281‐sup‐0001‐SuppMat.docx.

## Data Availability

The data that support the findings of this study are available from the corresponding author upon reasonable request. The codes used for the active learning framework and analysis in this work are publicly in the GitHub repository at https://github.com/minhee2043/AL_OER. Additional information regarding code implementation is available from the corresponding author upon reasonable request.
