# Supplementary material for: Accelerated Discovery of Earth‐Abundant Oxygen Evolution Catalysts Through Surface‐Informed Active Learning
Source: Adv Sci (Weinh). 2026 Oct 22:e78281. Online ahead of print. doi: 10.1002/advs.78281 (PMC13691240; doi:10.1002/advs.78281)
Supplement: Supplementary file 1 [file ADVS-9999-e78281-s001.docx]

**Supporting Information**

**Accelerated discovery of earth-abundant oxygen evolution catalysts through surface-informed active learning**

Hayun Jeon ^a,^‡, Minhee Park ^b,^‡, Won Bo Lee ^b^, Seyoung Park ^c^, YongJoo Kim ^d,*^, Sungwook Mhin ^c,*^

^a^Department of Advanced Materials Engineering, Kyonggi University, Gyeonggi 16227, Republic of Korea

^b^School of Chemical and Biological Engineering, Institute of Chemical Processes, Seoul National University, Seoul 08826, Republic of Korea

^c^Department of Energy and Materials Engineering, Dongguk University-Seoul, Seoul 04620, Republic of Korea

^d^Department of Materials Science and Engineering, Korea University, Seoul 02841, Republic of Korea

‡ These authors contributed equally to this work.
* Corresponding author’s E-mail addresses:

cjyjee@korea.ac.kr (Y. Kim)

swmhin@dongguk.edu (S. Mhin)

**1. Optimal energy in oxygen evolution reaction**

In this study, we calculate the stability of the intermediates *O and *OH on Ni(111), Fe(111), and Co(111) surfaces. Reaction energies were calculated from total energy calculations of relaxed surface slabs using equations 1-2.
 ${\Delta E}_{O}=E_{O}^{*}+E_{H_{2}}-E^{*}-E_{H_{2}O}$	(1)

${\Delta E}_{OH}=E_{OH}^{*}+\frac{1}{2}E_{H_{2}}-E^{*}-E_{H_{2}O}$	(2)

In the above equations, $E^{*}$ is the total energy of the clean surface, whereas $E_{O}^{*}$ and $E_{OH}^{*}$ denote the energy of the surface with O and OH adsorbed on it, respectively. $E_{H_{2}}$ and $E_{H_{2}O}$ are the total energies of the hydrogen and water molecule in vacuum. Note that electrons and protons released in the reaction are considered as hydrogen. With this choice, all reaction energies are referenced to the standard hydrogen electrode (SHE). To convert the reaction energies to free energies, we calculate free energies of the intermediates at zero potential and pH = 0 as $\Delta G=\Delta E+\Delta ZPE-T\Delta S$, where *∆E* is the reaction energy, $\Delta ZPE$ is the difference in zero-point energies due to the reaction, and *∆S* is the change in entropy. The zero-point energy and entropy changes as given by Nørskov et al. were added [1]. The values used are shown in Table S1. It is assumed that the zero-point energy and entropy changes are surface independent as for the zero-point energy change, previous works justified similar parameter using both restricted and unrestricted DFT [2-4]. The entropy change is strongly dominated by the gas phase reactants and products and can therefore also be assumed surface independent.
Therefore, the free energies can be represented with reaction energies as below:
 $\Delta G_{O}= \Delta E_{O}+0.05$	(3)

$\Delta G_{OH}= \Delta E_{OH}+0.35$	(4)

Replacing $\triangle E_{O}$ and $\triangle E_{OH}$ in equations 3-4 with equations 1-2, the final optimal energy for $E_{O*}-E_{OH*}$ can be converted as below:
 $\Delta G_{O}-\Delta G_{OH}= E_{O}^{*}-E_{OH}^{*}+$ $\frac{1}{2}$ $E_{H_{2}}-0.3 eV$	(5)

$\Delta G_{O}-\Delta G_{OH}= E_{O}^{*}-E_{OH}^{*}-3.7 eV$	(6)

Therefore, $E_{O}^{*}-E_{OH}^{*}$ should be 5.3 eV to achieve 1.6 eV, which is the optimal value by the scaling relations, i.e. 3.2/2 = 1.6 eV.^[3]^

**2. Active Learning Process**
The design space is constructed by examining all possible configurations of a 15-dimensional vector. Within groups 1, 3, 4, and 5, atoms are transformed into ensembles following patterns of [3,0,0], [2,1,0], ..., [0,0,3], while group 2, containing six atoms, follows patterns of [6,0,0], [5,1,0], [4,2,0], ..., [0,0,6]. Since multiple configurations can yield identical metal distributions within groups despite different atomic locations, multiplicities must be calculated. For a given group with total atoms n and metal distributions $n_{i}$, the multiplicity M is expressed as:
 $M= \frac{n!}{\prod_{i=1}^{3} (n_{i}!)}$	(7)

The total multiplicity for each configuration is determined by multiplying the individual multiplicities of all five groups. This procedure generates approximately 280,000 possible 15-dimensional vectors. However, due to the periodic boundary conditions inherent in plane-wave DFT calculations, not all these vectors correspond to viable 2×2×4 supercell models. Consequently, a subset of DFT-calculatable configurations is identified to define the space where the Expected Improvement (EI) function will select candidates for subsequent DFT calculations.

To predict the total energies for surfaces with *O and *OH adsorbates, we developed two independent Gaussian Process Regressor (GPR) models. These models utilize feature vectors representing the atomic structures of active sites, as illustrated in Figure 2a. Based on the assumption that adsorption energies vary continuously with respect to the feature vector, we employed the radial basis function (RBF) kernel:
 $k\left( x_{1}, x_{2} \right)=\sigma_{s}^{2}exp\left[ -\frac{\left| x_{1}-x_{2} \right|^{2}}{2l^{2}} \right]$	(8)

where $\sigma_{s}$ represents the signal level and l denotes the length scale. To account for statistical noise and prevent overfitting, we augmented the kernel with a white noise term, incorporating the noise variance $\sigma_{n}^{2}$ as a learnable parameter, as follows:
 $k\left( x_{1}, x_{2} \right)=\sigma_{s}^{2}exp\left[ -\frac{\left| x_{1}-x_{2} \right|^{2}}{2l^{2}} \right]+\sigma_{n}^{2}\delta_{12}.$	(9)

The model input consists of a 15-dimensional vector representing the surrounding atomic structure of the adsorbate:

$$\vec{x}=\left[ n_{Ni}^{1},n_{Fe}^{1},n_{Co}^{1},n_{Ni}^{2},n_{Fe}^{2},n_{Co}^{2},n_{Ni}^{3},n_{Fe}^{3},n_{Co}^{3},n_{Ni}^{4},n_{Fe}^{4},n_{Co}^{4},n_{Ni}^{5},n_{Fe}^{5},n_{Co}^{5} \right]$$

where each element represents the number of Ni, Co, and Fe atoms in the five neighboring groups around the adsorbate. This data space forms a hyperplane in 15 dimensions, satisfying the constraint $\sum_{i=1}^{15} x_{i}=18$. During each iteration cycle, the $E_{O*}$ and $E_{OH*}$ models are independently trained using DFT-calculated energies. Model parameters are optimized using the quasi-Newton L-BFGS-B algorithm. Each model contains 17 learnable parameters: signal variance $\sigma_{s}^{2}$, noise variance $\sigma_{n}^{2}$, and 15 length scale corresponding to each metal element across the five groups. To ensure optimal parameter selection, the optimization process is repeated ten times with different initial conditions to maximize the log-marginal likelihood.

The trained GPR model provided predictions of the mean and standard deviation of adsorption energies of all active sites within the ternary alloy search space. The mean represents the predicted adsorption energy, and the standard deviation indicates the uncertainty associated with this prediction. Consequently, the model can enhance the learning process by focusing on calculating additional information on the surface motifs that exhibit high uncertainty. In each active learning iteration, after training the two GPR models for the prediction of $E_{O}^{*}$ and $E_{OH}^{*}$, we employed expected improvement (EI) acquisition function to classify the candidate points in the data space that requires DFT-calculation. The EI is a widely used acquisition function that effectively balances the magnitude of exploration and exploitation. We adapted this function to prioritize the exploration of surfaces with adsorption energies close to the optimal values and substantial uncertainty. Here, we outline the general approach as utilized in this work.
The active learning procedure begins with DFT calculations of $E_{O}^{*}$ and $E_{OH}^{*}$ for 20 randomly selected NiCoFe alloy surfaces. These surfaces are converted to 15-dimensional vectors, and the GPR models are trained to predict properties across the entire design space. The Expected Improvement (EI) values are calculated for all DFT-calculatable vectors using the algorithm presented in Figure S3. To target surfaces with an optimal $E_{O}^{*}-E_{OH}^{*}$ difference of 5.3 eV, we modified the acquisition function to consider the difference between predicted means of $E_{O}^{*}$ and $E_{OH}^{*}$, while using the averaged standard deviations from both models to represent uncertainty. Through this approach, we can consider both models' uncertainty simultaneously while efficiently searching for surfaces that are likely to show the optimal $E_{O}^{*}-E_{OH}^{*}$value of 5.3 eV.

Following EI calculation, the 20 feature vectors with the highest EI values are converted to $2\times2\times4$ supercell structures. These structures undergo DFT calculations with *O and *OH adsorbates to determine $E_{O}^{*}$ and $E_{OH}^{*}$. The new results are incorporated into the training dataset for subsequent GPR model iterations. This process was continued for 15 cycles, until the prediction errors converged (Figure S6). Of the 300 configurations selected, in 46 cases the O and OH adsorbates relaxed to different surface sites, so the pair could not be compared and was excluded, leaving 254 configurations, divided into 190 training and 64 test surfaces.

The batch size of 20 was chosen for computational throughput, allowing 40 slab relaxations to be run concurrently per cycle. Replaying the procedure over the completed dataset with batch sizes from 1 to 40 changes the descriptor error at a fixed budget of 100 DFT calculations by less than 0.01 eV (Figure S4), and configurations selected within a batch retain largely independent information (Figure S5), confirming that this choice does not compromise sampling efficiency.

**3. Activity Calculation and Optimization**

The predicted ${\Delta G}_{O}-{\Delta G}_{OH}$ values of the active sites on the catalyst surface were combined with the likelihood of surface generation to evaluate the activity of the catalyst of given atomic ratio. We used an expression based on the Sabatier volcano, in which the catalytic activity was expressed as follows:

$A=\sum_{i=1}^{Z} \left( \prod_{k}^{metals} f_{k}^{n_{ik}} \right)\exp\left( -\frac{\left| \Delta E_{i}-\Delta E_{opt} \right|}{k_{B}T} \right)$	(10)

Here, *Z* represents the total count of local atomic environments, $f_{k}$ is the atomic ratio of the metal element $k$, and $n_{ik}$ denotes the number of atoms of element $k$ in the active site $i$. As a result, the term in product notation signifies the likelihood of encountering a particular binding site $i$. $\Delta E_{i}$ represents the predicted ${\Delta E}_{O}-{\Delta E}_{OH}$ of the surface configuration, while $\Delta E_{opt}=$1.9 eV represents the desired energy value as per the scaling relations of OER. Finally, $k_{B}$ denotes Boltzmann’s constant; T was set to 300 K. The activity function comprises two components: the probability of a specific binding site and the disparity between the optimal and modeled adsorption energies. The contributions from each site were aggregated to determine the total activity of a specific atomic fraction. Sequential least squares programming was utilized to determine the fraction of each metal that maximized the catalytic activity.

**Figure S1. Parity plots comparing DFT-calculated and active learning-predicted adsorption energies on the test set.** Comparison of predicted versus DFT energies for $E_{O}^{*}$ (a, c, e, g, i, k, m, o) and $E_{OH}^{*}$ (b, d, f, h, j, l, n, p) predictions using different active learning models: (a, b) GPR, (c, d) Random Forest Regression (RFR), (e, f) Linear Regression (LR), (g, h) Gradient Boosting Regression (GBR), (i, j) k-Nearest Neighbors Regression (KNR), (k, l) AdaBoost Regression (ABR), (m, n) Support Vector Regression (SVR), and (o, p) Bagging Regression (BAGR).

**Figure S2. Performance comparison of active learning models for adsorption energy prediction.** (a) Mean Absolute Error (MAE), (b) Root Mean Squared Error (RMSE), and (c) R² score for $E_{O}^{*}$ (teal bars) and $E_{OH}^{*}$ (orange bars) predictions across eight active learning models: GPR, LR, KNR, SVR, RFR, GBR, ABR, and BAGR. Lower MAE and RMSE values indicate better prediction accuracy, while R² scores closer to 1.0 (dashed line in panel c) indicate better model fit. GPR shows the best overall performance with the lowest errors and highest R² scores for both adsorption energies.

Figure S3. Modified Expected Improvement Algorithm. The full algorithm of the modified expected improvement acquisition function employed in this work. μ(x) is the difference between the two means obtained from the two GPR models at point x. σ(x) is the average of the two standard deviations from the two GPR models at point x. Φ and ϕ indicate the CDF (cumulative distribution function) and PDF (probability density function) of the standard normal distribution. The parameter ξ determines the amount of exploration and increasing its value raises the potential improvements in regions of high uncertainty. In this study, ξ is set to recommended default value 0.01. Unlike the usual acquisition function used for Bayesian optimization, η denotes the optimal value to increase the accuracy for the near-optimal search space.

Figure S4. Effect of the acquisition batch size on data efficiency. (a) Mean absolute error of the OER descriptor $\boldsymbol{E}_{\boldsymbol{O}}^{\boldsymbol{*}}\mathbf{-}\boldsymbol{E}_{\boldsymbol{OH}}^{\boldsymbol{*}}$ on a fixed held-out test set as a function of the number of DFT calculations, for batch sizes of 1, 5, 10, 20 and 40. The active learning procedure was replayed over the completed dataset with 8 independent random initializations of the 20-surface seed set. (b) Descriptor error at a fixed budget of 100 DFT calculations as a function of batch size. The dotted line marks the value of 20 used in this work. Lines and points are medians and shaded bands and error bars are interquartile ranges over the 8 initializations.

**Figure S5. Information overlap within an acquisition batch.** Fraction of the Gaussian process predictive variance at the k-th selected configuration already accounted for by selections 1 to k−1 of the same batch, computed with fixed kernel hyperparameters across all 15 cycles and both models (n = 28 per point). Values are shown to k = 13, beyond which fewer cycles contain that many paired configurations. Line: median; shaded band: interquartile range.

**Figure S6. Convergence of prediction errors during iterative active learning.** RMSE as a function of active learning iterations for $E_{O}^{*}$ (blue circles), $E_{OH}^{*}$ (orange squares), and the adsorption energy difference $E_{O}^{*}-E_{OH}^{*}$ calculated by two methods: direct prediction from the difference of predicted values (green triangles) and error propagation estimation (red inverted triangles). The direct method calculates the difference using predicted individual energies, while the propagated error represents the theoretical upper bound assuming independent errors ($\sqrt{{RMSE}_{O*}^{2}+{RMSE}_{OH*}^{2}}$).

Figure S7. Distribution of GPR predicted OER descriptor values across the catalyst search space. Histogram showing the frequency distribution of $\boldsymbol{\Delta}\boldsymbol{G}_{\boldsymbol{O}}\boldsymbol{-\Delta}\boldsymbol{G}_{\boldsymbol{OH}}$ values for the entire NiCoFe surface dataset. The distribution is centered near 0 eV with a right-skewed tail extending to higher values. The optimal value is 1.6 eV, which corresponds to minimizing the overpotential in the oxygen evolution reaction.

Figure S8. Data efficiency of the active-learning framework. (a) MAE of the OER descriptor $\boldsymbol{E}_{\boldsymbol{O}}^{\boldsymbol{*}}\mathbf{-}\boldsymbol{E}_{\boldsymbol{OH}}^{\boldsymbol{*}}$ on the held-out test set versus the number of DFT calculations; dotted line, constant predictor returning the training-set mean. (b) The same, for 30 random initializations of the seed set (thin lines) and their mean (black). (c) Global test error and (d) simple regret for the modified EI acquisition and random selection at matched budget. Lines are means over 30 repeats; shaded bands, ±1 standard deviation.

**Figure S9. Targeted accuracy of the EI acquisition.** MAE for the modified EI acquisition and random selection, restricted to the most promising decile of the test set (the 10% of configurations with the largest $E_{O}^{*}-E_{OH}^{*}$). Lines are means over 30 repeats; shaded bands, ±1 standard deviation.

Figure S10. Distribution of DFT-calculated adsorption free energies for surfaces sampled during active learning. Histogram showing the frequency distribution of (a) $\boldsymbol{\Delta}\boldsymbol{G}_{\boldsymbol{O}}$ and (b) $\boldsymbol{\Delta}\boldsymbol{G}_{\boldsymbol{OH}}$ values obtained from DFT calculations on catalyst surfaces selected through the active learning.

Figure S11. Distribution of predicted OER descriptor values for the optimal catalyst Ni₅₅Co₁₀Fe₃₅. Histogram showing the percentage distribution of $\boldsymbol{E}_{\boldsymbol{O}}^{\boldsymbol{*}}\mathbf{-}\boldsymbol{E}_{\boldsymbol{OH}}^{\boldsymbol{*}}$ values across all possible surface configurations of the top-performing catalyst composition Ni₅₅Co₁₀Fe₃₅ identified through AL process.

**Figure S12. Tafel slopes for Ni_x_**-**Co_y_**-**Fe_(100-x-y)_ alloys with FCC structure.**

**Figure S13. XRD patterns of Ni_x_**-**Co_y_**-**Fe_(100-x-y)_ alloys with FCC structure.**

**Figure S14. XPS spectra of NCF6: Ni 2p, Co 2p, and Fe 2p.**

**Figure S15. XPS spectra of NCF6: C 1s and O 1s.**

**Figure S16. Surface analysis for confirming the formation of alloy in NCF6.** (a) TEM images for NCF6. (b) Elemental mapping image for C, O, Ni, Co, and Fe in NCF6.

**Figure S17. XPS spectra of NiFe: Ni 2p and Fe 2p.**

|  | TS | T$\boldsymbol{\Delta S}$ | ZPE | $\boldsymbol{\Delta ZPE}$ | $\boldsymbol{\Delta ZPE-T\Delta S}$ |
| --- | --- | --- | --- | --- | --- |
| H_2_O | 0.67 | 0 | 0.56 | 0 | 0 |
| *OH + 1/2H_2_ | 0.20 | -0.47 | 0.44 | -0.12 | 0.35 |
| *O+H_2_ | 0.41 | -0.27 | 0.34 | -0.22 | 0.05 |
| 1/2O_2_+H_2_ | 0.73 | 0.05 | 0.32 | -0.24 | -0.29 |

**Table S1. Entropic and Zero-Point energy contributions to the Free energies of gaseous and adsorbed OER species as calculated by Nørskov et al. in eV.^[1]^**

| Metal | Ontop | Bridge | FCC hollow |
| --- | --- | --- | --- |
| Ni | 2.1235 | 0.759 | 0.111 |
| Co | 1.529 | 0.188 | -0.333 |
| Fe | 0.729 | -0.465 | -0.759 |

**Table S2. *O adsorption energies at different adsorption sites of pure metal surfaces in eV**

| Metal | Ontop | Bridge | FCC hollow |
| --- | --- | --- | --- |
| Ni | 0.615 | 0.241 | 0.230 |
| Co | 0.460 | 0.041 | -0.0555 |
| Fe | 0.0998 | -0.218 | -0.494 |

**Table S3. *OH adsorption energies at different adsorption sites of pure metal surfaces in eV**

| Iteration | $\boldsymbol{l}_{\boldsymbol{*O}}$ | $\boldsymbol{\sigma}_{\boldsymbol{*O}}$ | $\boldsymbol{l}_{\boldsymbol{*OH}}$ | $\boldsymbol{\sigma}_{\boldsymbol{*OH}}$ |
| --- | --- | --- | --- | --- |
| 1 | 1.091 | 0.000 | 7.265 | 0.000 |
| 2 | 0.411 | 0.000 | 1.091 | 0.000 |
| 3 | 70.457 | 0.008 | 7.184 | 0.000 |
| 4 | 1.091 | 0.000 | 4.571 | 0.001 |
| 5 | 27.294 | 0.000 | 7.184 | 0.000 |
| 6 | 1.146 | 0.000 | 67.322 | 0.011 |
| 7 | 1.146 | 0.000 | 7.159 | 0.000 |
| 8 | 7.184 | 0.000 | 4.447 | 0.002 |
| 9 | 0.976 | 0.041 | 20.679 | 0.001 |
| 10 | 0.976 | 0.041 | 20.678 | 0.001 |
| 11 | 0.075 | 0.057 | 20.678 | 0.001 |
| 12 | 7.159 | 0.000 | 7.184 | 0.000 |
| 13 | 2.637 | 0.000 | 4.571 | 0.001 |
| 14 | 4.571 | 0.001 | 4.571 | 0.001 |
| 15 | 0.411 | 0.000 | 1.013 | 0.003 |

**Table S4. Optimized length scale and noise level hyperparameters of the model at each iteration loop.**

|  | Ni | Co | Fe | Activity |
| --- | --- | --- | --- | --- |
| #1 | 55 | 10 | 35 | 0.00966 |
| #2 | 50 | 15 | 35 | 0.00945 |
| #3 | 60 | 10 | 30 | 0.00934 |
| #4 | 45 | 15 | 40 | 0.00917 |
| #5 | 50 | 10 | 40 | 0.00914 |
| #6 | 60 | 5 | 35 | 0.00905 |

**Table S5. Optimal compositions obtained from the active learning model using 5 at. % grid search**

| Metal | $\mathbf{E}_{\mathbf{O}}^{\mathbf{*}}$ | $\mathbf{E}_{\mathbf{OH}}^{\mathbf{*}}$ | $\mathbf{E}_{\mathbf{O}}^{\mathbf{*}}\mathbf{-}\mathbf{E}_{\mathbf{OH}}^{\mathbf{*}}$ |
| --- | --- | --- | --- |
| Ni | -80.549 | -83.918 | 3.369 |
| Co | -102.043 | -105.254 | 3.211 |
| Fe | -120.210 | -123.434 | 3.224 |

**Table S6. The total energies (in eV) of the pure metal surface with adsorbed O and OH, as well as the energy difference between these two states.**

|  | **Composition** |
| --- | --- |
| **NCF 1** | Ni_30_-Co_10_-Fe_60_ |
| **NCF 3** | Ni_33_-Co_33_-Fe_33_ |
| **NCF 5** | Ni_30_-Co_60_-Fe_10_ |
| **NCF 6** | Ni_55_-Co_10_-Fe_35_ |
| **NCF 7** | Ni_75_-Co_15_-Fe_10_ |
| **NCF 8** | Ni_60_-Co_30_-Fe_10_ |
| **NCF 9** | Ni_15_-Co_75_-Fe_10_ |
| **NCF 10** | Ni_15_-Co_05_-Fe_80_ |
| **NCF 11** | Ni_25_-Co_05_-Fe_70_ |
| **NCF 12** | Ni_10_-Co_25_-Fe_65_ |
| **NCF 13** | Ni_15_-Co_40_-Fe_45_ |
| **NCF 14** | Ni_30_-Co_25_-Fe_45_ |
| **NCF 15** | Ni_15_-Co_55_-Fe_35_ |

**Table S7. Composition of Ni_x_**-**Co_y_**-**Fe_(100-x-y)_ alloys.**

| **Core level (region)** | **Species** | **Area (a.u.)** | **Fraction (%)** |
| --- | --- | --- | --- |
| **Ni 2p_3/2_ region** | Ni^0^ | 1655.57 | 70.46 |
|  | Ni^2+^ | 171.35 | 7.29 |
|  | Ni^3+^ | 522.87 | 22.25 |
| **Co 2p_3/2_ region** | Co^0^ | 35.06 | 6.67 |
|  | Co^2+^ | 442.88 | 84.27 |
|  | Co^3+^ | 47.62 | 9.06 |
| **Fe 2p_3/2_ region** | Fe^0^ | 74.59 | 9.04 |
|  | Fe^2+^ | 266.43 | 32.29 |
|  | Fe^3+^ | 484.01 | 58.67 |

**Table S8.** **Integrated XPS peak areas and relative fractions of M⁰, M²⁺, and M³⁺ species of NCF6 obtained from the deconvolution of Ni 2p, Co 2p, and Fe 2p spectra.** The peak areas were calculated after background subtraction, and the relative fractions were determined using the main fitted components corresponding to each oxidation state, excluding satellite contributions.

| **Catalysts** | **η_10_ (mV vs. RHE)** | **Tafel slope**  **(mV dec^-1^)** | **Reference** |
| --- | --- | --- | --- |
| **Ni_55_Co_10_Fe_35_** | 327 | 43.07 | This work |
| **Ni-W-B alloy** | 334 | 52 | [5] |
| **GCE/TRuCoTPyPz** | 447 | 75 | [6] |
| **Mo_2_C-CoNi@CNFs** | 306 | 72.4 | [7] |
| **CoNiCuMnFe/C** | 350 | 79 | [8] |
| **FeCoNiCu** | 358 | 75.69 | [9] |
| **La(CrMnFeCo_2_Ni)O_3_** | 325 | 51.2 | [10] |
| **FeNiCoCuMo HEA** | ~380 | ~77 | [11] |
| **PtNiCuMnMo**  **high-entropy alloy** | 250 | 124 | [12] |

**Table S9. Comparison of OER performance of reported catalysts under various electrolyte conditions.**

| **Core level (region)** | **Species** | **Area (a.u.)** | **Fraction (%)** |
| --- | --- | --- | --- |
| **Ni 2p_3/2_ region** | Ni^0^ | 1753.03 | 75.38 |
|  | Ni^2+^ | 402.83 | 17.32 |
|  | Ni^3+^ | 169.74 | 7.3 |
| **Fe 2p_3/2_ region** | Fe^0^ | 1537.94 | 57.56 |
|  | Fe^2+^ | 584.28 | 21.87 |
|  | Fe^3+^ | 549.61 | 20.57 |

**Table S10.** **Integrated XPS peak areas and relative fractions of M⁰, M²⁺, and M³⁺ species of NiFe obtained from the deconvolution of Ni 2p and Fe 2p spectra.** The peak areas were calculated after background subtraction, and the relative fractions were determined using the main fitted components corresponding to each oxidation state, excluding satellite contributions.

**References**

[1] J. K. Nørskov et al., “Origin of the overpotential for oxygen reduction at a fuel-cell cathode,” *Journal of Physical Chemistry B* **108** (2004): 17886–17892.

[2] P. Liao, J. A. Keith, and E. A. Carter, “Water oxidation on pure and doped hematite (0001) surfaces,” *Journal of the American Chemical Society* **134** (2012): 13296–13309.

[3] I. C. Man et al., “Universality in oxygen evolution electrocatalysis on oxide surfaces,” *ChemCatChem* **3** (2011): 1159–1165.

[4] A. Valdes et al., “Oxidation and photo-oxidation of water on TiO2 surfaces,” *Journal of Physical Chemistry C* **112** (2008): 9872–9879.

[5] S. Li et al., “Ni–B nanostructured electrocatalysts with high efficiency for oxygen evolution prepared through selective dealloying from Ni–W–B amorphous alloy,” *International Journal of Hydrogen Energy* **68** (2024): 614–622.

[6] P. M. Viyanni and M. G. Sethuraman, “Influence of diverse hydrogen bond donors in deep eutectic solvents on the electrochemical deposition of NiCu alloy on stainless steel mesh: Exploring NiCu efficacy in water splitting and methanol oxidation reactions,” *International Journal of Hydrogen Energy* **130** (2025): 615–630.

[7] Y. Ding et al., “Mo2C–CoNi heterostructure-knotted CNFs for efficient hydrogen and oxygen evolution reaction,” *International Journal of Hydrogen Energy* **102** (2025): 866–873.

[8] Z. Wang et al., “Flower-sphere CoNiCuMnFe/C high-entropy alloy catalysts for enhanced oxygen evolution reaction kinetics,” *Journal of Alloys and Compounds* **1026** (2025): 180421.

[9] Q. Chen et al., “Noble metal-free XFeCoNiCu (X = Cr, Mg, and Mn) high entropy alloys for efficient ORR/OER bifunctional catalysis,” *Colloids and Surfaces A: Physicochemical and Engineering Aspects* **709** (2025): 136106.

[10] M. He et al., “Lanthanum-based nanomaterials for oxygen evolution reaction,” *Journal of Rare Earths* **43** (2025): 1091–1099.

[11] V. M. Jiménez-Arévalo et al., “Energy transfer by mechanical alloying and electrocatalytic performance of the as-sintered self-supported high-entropy alloy FeNiCoCuMo,” *Advanced Engineering Materials* **27** (2024): 2400807.

[12] F. Miao et al., “A dual-phase PtNiCuMnMo high-entropy alloy as high-performance electrocatalyst for oxygen evolution reaction,” *Applied Surface Science* **684** (2025): 161865.
